# What the radiologist needs to know about restaging of rectal carcinoma after chemoradiation therapy

**DOI:** 10.1186/1470-7330-14-S1-P16

**Published:** 2014-10-09

**Authors:** ME Nazar, F Grana, L Alarcon, G Caimi Romero, N Rotholtz, EP Eyheremendy

**Affiliations:** 1 German Hospital, Buenos Aires, Argentina

## Aim

The purpose of this exhibit is to describe and compare the high-resolution MRI features of rectal carcinoma after chemoradiation treatment (CRT) and to correlate with the histologic findings after total mesorectal excision (TME).

## Method

High resolution T2-W MR imaging (HRMRI) was performed in a 1.5 T unit between January 2013 and February 2014 before and immediately after CRT in the care of 25 patients with locally advanced adenocarcinoma of the rectum. After total mesorectal excision (TME) the piece was cut by the pathologist under the supervision of the radiologist, who indicated areas of residual tumour after neoadjuvant therapy or changes such as fibrosis, oedema, cellular and acellular mucin, desmoplastic reaction and pseudotumour appearance. Thus, initially we did a correlation between the macroscopic and MR imaging. Subsequently, we performed the same correlation but in this case between microscopy and MR imaging. Changes in morphologic and signal intensity features were evaluated with respect to primary tumour and nodal downstaging.

## Summary

Emerging evidence has shown the prognostic importance of reassessing rectal cancer using HRMRI after completion of CRT. A systematic cooperation between radiologist and pathologist is essential for optimal treatment planning and patient care.

